# Review of cardiovascular magnetic resonance in human immunodeficiency virus-associated cardiovascular disease

**DOI:** 10.4102/sajr.v21i2.1248

**Published:** 2017-11-14

**Authors:** Vishesh Sood, Stephen Jermy, Hadil Saad, Petronella Samuels, Sulaiman Moosa, Ntobeko Ntusi

**Affiliations:** 1 Division of Radiology, Department of Radiation Medicine, University of Cape Town; 2 Groote Schuur Hospital, Cape Town, South Africa; 3 Cape Universities Body Imaging Centre, University of Cape Town, South Africa; 4 Department of Medicine, University of Cape Town, South Africa; 5 Hatter Institute of Cardiovascular Research in Africa, Department of Medicine, University of Cape Town, South Africa

## Abstract

Despite ongoing advances in the treatment of patients with human immunodeficiency virus (HIV) or acquired immunodeficiency syndrome (AIDS), they remain a major global public health concern conferring an increased risk of morbidity and mortality in affected individuals. This is, in part, because of the widespread dysfunction imposed by HIV and its treatment on the cardiovascular system, including the myocardium, valvular apparatus, pericardium and coronary, pulmonary and peripheral vasculature. In recent times, cardiovascular magnetic resonance (CMR) imaging has emerged as the gold standard tool for assessment of a variety of indications, allowing comprehensive characterisation of functional, morphological, metabolic and haemodynamic sequelae of several cardiovascular pathologies. Furthermore, continued advancement in imaging techniques has yielded novel insights into the underlying pathophysiology and guides future therapeutic strategies. In this article, we review the various clinical phenotypes of HIV-associated cardiovascular disease and highlight the utility of CMR in their assessment.

## Introduction

As of late 2015, there were approximately 36.7 million people worldwide living with human immunodeficiency virus (HIV) or acquired immunodeficiency syndrome (AIDS), with an estimated 2.1 million people becoming newly infected in that same year.^1^ Despite the decreasing incidence of *de novo* infections seen over the past decade, HIV infection remains a major global public health concern, with most infected individuals living in low- to middle-income countries, particularly in sub-Saharan Africa.^1^

As a consequence of the introduction of combination antiretroviral therapy (ART), the survival of people living with HIV infection has improved substantially.^2^ South Africa has the largest number of people living with HIV infection and also the largest ART roll-out programme globally.^3^ However, despite our improved understanding of the disease and early initiation of ART, HIV infection is associated with significant complications, including cardiovascular disease (CVD). HIV-associated CVD results from a complex interplay of factors, including, but not limited to, chronic inflammatory processes resulting from HIV infection itself, opportunistic infections, concurrent metabolic changes resulting from the use of ART and conventional risk factors for atherosclerotic CVD.^4^ Consequently, HIV-associated CVD involves all segments of the cardiovascular tree, commonly affecting the left ventricular (LV) myocardium, valvular apparatus, pericardium and coronary, pulmonary and peripheral vasculature.^4^

Various modalities of cardiovascular imaging are integral to the assessment of CVD, and many are ingrained into the modern practice of cardiovascular medicine. Cardiovascular magnetic resonance (CMR) has emerged as the gold standard technique for many indications, allowing comprehensive characterisation of functional, morphological, metabolic and hemodynamic sequelae of various cardiovascular manifestations. In this article, we review phenotypes of HIV-associated CVD along a clinical continuum and highlight the utility of CMR in their assessment.

## Cardiovascular magnetic resonance techniques

Several unique properties of CMR contribute to its widespread utility in the assessment of the cardiovascular system. The relatively high spatial and temporal resolution coupled with excellent tissue contrast enables comprehensive assessment of multiple parameters pertaining to cardiovascular morphology and function, without exposure to ionising radiation.^5^ Furthermore, the ability to obtain images in any tomographic plane regardless of body habitus confers significant advantage in patients with limited sonographic acoustic windows.^5^

Characterisation of myocardial tissue is a unique feature of CMR, traditionally achieved through late gadolinium-enhanced imaging and based on the relative difference in volume of distribution of intravenously administered contrast [and subsequent alteration of longitudinal relaxation (T1) times] between normal and abnormal myocardium.^5^ More recently, native (precontrast) T1 and T2 mapping techniques have allowed direct measurement of myocardial relaxation times on a pixel-wise basis, parameters which have been extensively validated offering similar diagnostic performance and superior sensitivity for inflammation, infiltration, acute injury and fibrosis as compared with delayed enhancement imaging in detecting myocardial pathology.^5,6,7^ T1 maps are most commonly acquired using modified look-locker inversion recovery (MOLLI)-based or saturation recovery single-shot acquisition (SASHA)-based sequences in the short-axis plane at the basal, mid and apical LV levels. The underlying principle involves the application of an inversion or saturation pulse followed by successive sampling of the relaxation curve as myocardial longitudinal magnetisation returns to its original level. The entire relaxation curve is then extrapolated from the acquired data and the native T1 values extracted. For ease of interpretation, these values are displayed as a colour map superimposed on anatomic images, allowing global and segmental quantification of T1 values using targeted regions-of-interest (ROIs). The further acquisition of T1 maps following contrast administration allows for the estimation of myocardial extracellular volume (ECV), a marker of myocardial tissue remodelling which has been shown to be a robust measure of the degree of myocardial fibrosis.^7^

**FIGURE 1:**
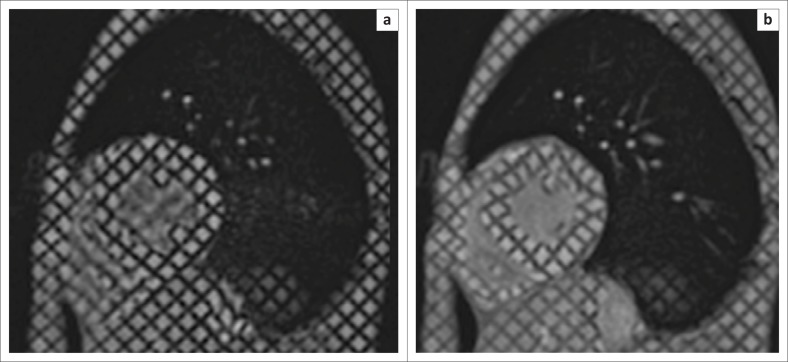
Short-axis images through the mid-left ventricle with application of myocardial tagging at end-diastole (a). Tag lines follow myocardial deformation (b) during the cardiac cycle and allow quantification of myocardial strain parameters in the circumferential, longitudinal and radial directions.

In addition to the assessment of biventricular function using ejection fraction (EF), several CMR techniques have been developed to quantify myocardial deformation (i.e. strain) throughout the cardiac cycle. In essence, this is achieved using multiple magnetic labels (in the form of black lines or tags) which are superimposed on and embedded into the myocardium at the start of a cine sequence (see Figure 1). The subsequent deformation of these tags is assessed throughout the cardiac cycle and allows for inferences to be made about myocardial strain in various (circumferential, radial and longitudinal) planes.

## Left ventricular dysfunction

Left ventricular systolic and diastolic dysfunction are well documented in HIV-infected persons, with most data emerging from echocardiography-based studies.^8,9^ Conventional indices available for the assessment of LV function include the EF, volumes, wall motion, thickness and mass.^5^ The measurement of systolic and diastolic strain and strain rate parameters provides more sensitive information about global and regional cardiac function and cardiac deformation properties, and changes in strain parameters often predate the development of overt LV systolic dysfunction.^10^

Several studies using CMR have consistently demonstrated the presence of regional LV systolic and diastolic dysfunction in asymptomatic individuals.^10,11,12^ In the setting of preserved EF, impairment was detected on CMR as reduced circumferential,^10^ longitudinal^11^ and radial^12^ strain and strain rate measurements.

A cross-sectional study of 698 HIV-infected persons showed that 48% had diastolic dysfunction, which was associated with the co-existence of conventional cardiovascular risk factors (including age, elevated body mass index, elevated total cholesterol and presence of hypertension and diabetes mellitus).^13^ Another study of 129 HIV-infected patients showed a 47% higher median myocardial lipid content and 76% prevalence of myocardial fibrosis, using MR spectroscopic and parametric mapping techniques, respectively, raising suspicion that these morphologic abnormalities may underlie cardiac dysfunction in this subset of patients.^11^

## Myocarditis

Inflammatory changes within the myocardium have been extensively studied in the context of HIV infection and contribute significantly to the eventual decline in cardiovascular function and the development of cardiomyopathy in affected individuals.^4,14^ Direct invasion of cardiomyocytes by HIV has been described, though this occurs in a haphazard fashion with no clear association between viral load and extent of myocardial involvement.^15^ Worsening immunosuppression predisposes to cardiotropic viral infection (herpes simplex virus, cytomegalovirus, parvovirus and Coxsackie B3 most commonly implicated) and infection by opportunistic pathogens including *Mycobacterium tuberculosis, Toxoplasma gondii, Cryptococcus neoformans* and *Histoplasma capsulatum*.^4^ It is worth noting, however, that no specific pathogen is identified in up to 80% of affected patients, and clinical presentation is heterogeneous with a large proportion of patients remaining asymptomatic despite ongoing subclinical myocardial oedema and inflammation.^16^

Cardiovascular magnetic resonance is a powerful tool for diagnosing myocarditis because of its ability to accurately delineate areas of myocardial oedema, necrosis, infiltration and fibrosis. Standard cine balanced steady-state free precession (bSSFP) sequences may show regional wall motion abnormalities, areas of increased wall thickness associated with acute inflammation and the presence of co-existent pathologies, which includes characterisation of the extent, location and haemodynamic significance of any pericardial effusions.

**FIGURE 2:**
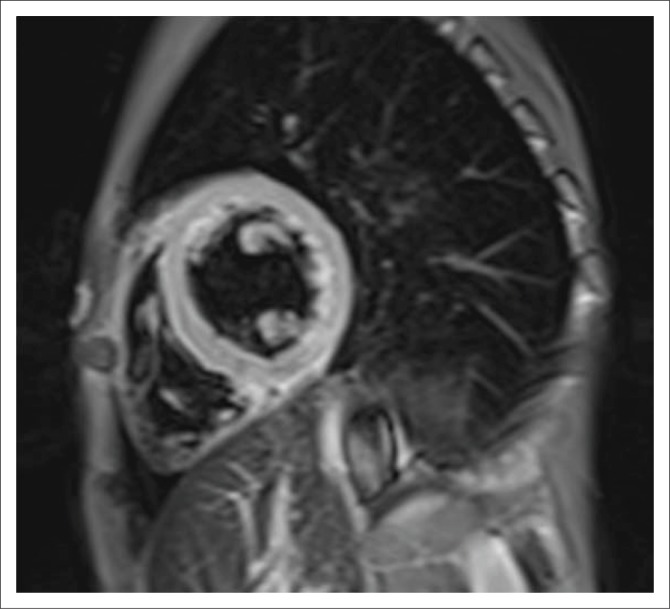
T2-weighted STIR image at the level of the mid-left ventricle shows an elevated myocardial skeletal muscle signal intensity ratio (SIR) of 2.5 (normal < 1.9) in keeping with diffuse myocardial oedema.

**FIGURE 3:**
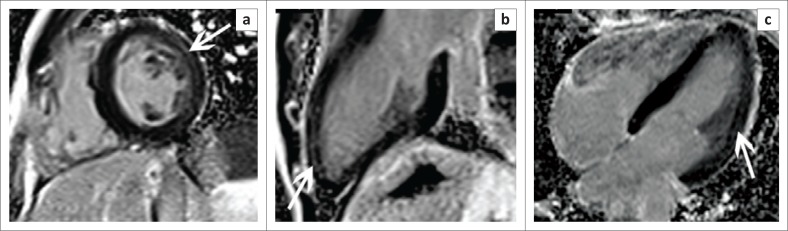
Short-axis (a), 2-chamber (b) and 4-chamber (c) late gadolinium phase-sensitive inversion recovery images show linear mid-wall enhancement in the lateral wall of the LV (white arrows).

Myocardial oedema appears as high signal on T2-weighted short tau inversion recovery sequences (STIR) (see Figure 2), and more recently, prolongation of native T1 and T2 values as detected through mapping techniques has been shown to be a sensitive imaging biomarker of active myocardial inflammation.^6^ The acquisition of post-contrast images yields further insight as regional vasodilation and acute myocardial necrosis result in altered gadolinium kinetics, with an increased uptake and rapid distribution of gadolinium chelates into the expanded interstitial space. The acquisition of T1-weighted images acquired approximately 2–5 min following contrast administration allows for the quantitative assessment of the myocardial early gadolinium enhancement ratio (EGEr), a ratio between myocardial and musculoskeletal enhancement, which highlights the variation in gadolinium distribution resulting from tissue damage (typically EGEr > 4.0 considered abnormal). Late gadolinium-enhanced MR images use inversion recovery sequences to null normal myocardium, highlighting areas of retained contrast as high signal areas representative of focal myocardial fibrosis. Findings classically include linear, patchy or nodular areas of enhancement in a non-coronary distribution (most commonly the basal inferolateral walls) with a mid-wall or sub-epicardial pattern^17^ (see Figure 3). Lastly, while more commonly a marker of diffuse myocardial fibrosis, the ECV fraction (calculated from native and post-contrast T1 maps) may also be elevated in myocarditis.^18^

Luetkens et al. studied 28 HIV-infected patients, on HAART with HIV RNA < 200 copies/mL, using CMR and found elevated parameters indicating myocardial inflammation (native T1 time, relative T2 signal intensity ratios and EGEr) as compared with healthy controls.^19^ Ntusi et al. looking at 103 HIV-infected individuals, without known CVD, found evidence of subclinical myocardial oedema and an increased incidence of pericardial effusions, providing additional evidence for chronic myocardial inflammation.^20^

## Cardiomyopathy

**FIGURE 4:**
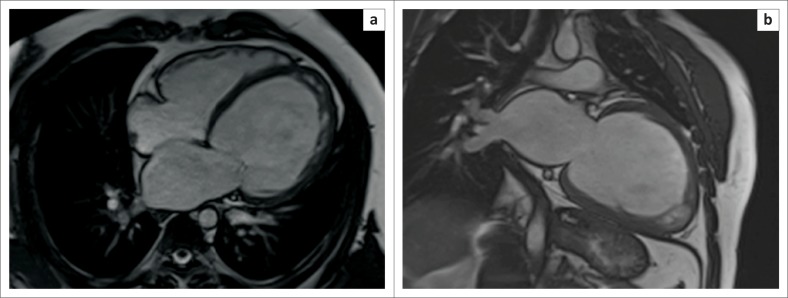
Four-chamber (a) and 2-chamber (b) SSFP images showing a markedly dilated left ventricle in a patient with known HIV-associated cardiomyopathy.

Acquired cardiomyopathy in the context of HIV infection represents the final common pathway of a complex and multifactorial process resulting in systolic and diastolic dysfunction and, most often, progression to a dilated cardiomyopathy (see Figure 4). In ‘The Heart of Soweto Study’ the prevalence of HIV-associated cardiomyopathy was reported as 38%, comprising both patients with clinical features of heart failure and those noted to have subclinical LV dysfunction on echocardiography.^21^

**FIGURE 5:**
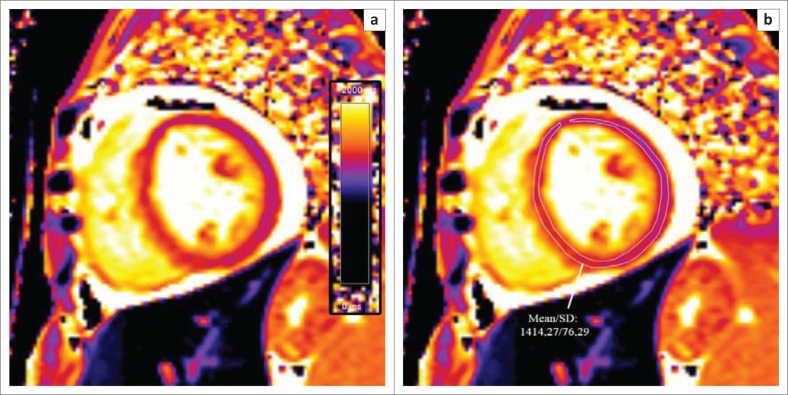
T1 map at the level of the mid-LV (a) shows a markedly elevated myocardial T1 time of 1414 m/s (normal 1052 m/s ± 23 m/s at 3 T) (b) in keeping with diffuse myocardial fibrosis.

Although echocardiography remains the first-line imaging modality for the diagnosis of HIV-associated cardiomyopathy, CMR allows comprehensive, easily reproducible assessment of cardiac morphology and function, with added tissue characterisation techniques allowing the detection and quantification of underlying myocardial fibrosis through late gadolinium enhancement (LGE) and parametric mapping techniques (see Figure 5). This proves useful in both prognostication and longitudinal follow-up in this subset of patients as higher levels of myocardial fibrosis have been correlated with an increase in all-cause mortality.

## Pericardial disease

The spectrum of pericardial disease associated with HIV is broad with isolated pericardial effusions reported as the commonest manifestation. Although non-specific, their presence supports active inflammation and may add weight to the diagnosis of subclinical myocarditis or point to primary pericardial disease, with *Mycobacterium tuberculosis* being the most likely pathogen implicated. Historic studies demonstrated that tuberculous pericarditis accounted for over 80% of causes of pericardial effusion in HIV-infected individuals occurring in sub-Saharan Africa.^22,23^ The underlying pathophysiology implicates retrograde lymphatic spread from peribronchial and mediastinal lymph nodes or haematogenous dissemination from primary tuberculous infection. The manifestations of tuberculous involvement of the pericardium may include pericarditis, pericardial effusions with or without associated effusive-constrictive syndromes and progression to calcified pericardial constriction.^24,25,26^

**FIGURE 6:**
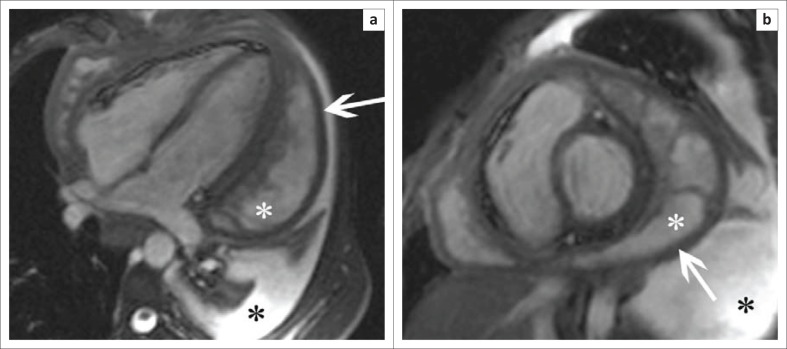
CMR SSFP images (a and b) show marked pericardial thickening (white arrows) and a large pericardial effusion containing organised fibrin strands (white asterisk). A large left-sided pleural effusion is also evident (black asterisk).

**FIGURE 7:**
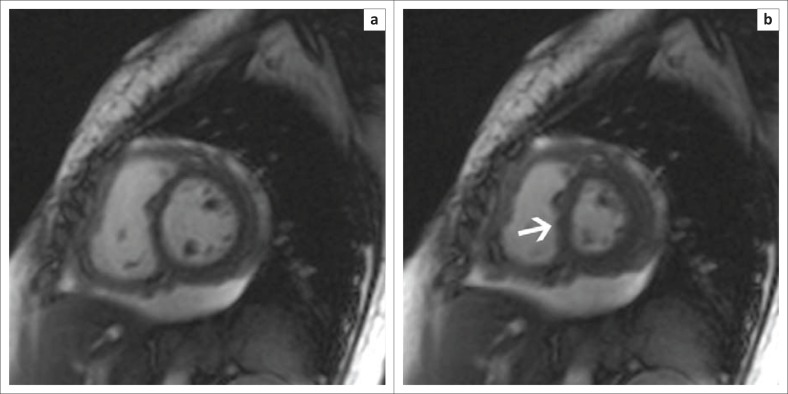
Short-axis SSFP sequences at end-diastole (left) and end-systole (right) show systolic flattening of the interventricular septum (white arrow) indicative of right ventricular pressure overload.

Cardiovascular magnetic resonance is useful in both characterisation of morphology and assessment of haemodynamic sequelae associated with tuberculous pericardial disease. Cine SSFP images demonstrate the size and location of effusions, and although not specifically T2 weighted, they frequently allow visualisation of organised fibrin strands within the pericardial sac (see Figure 6). Furthermore, as progressive inflammation results in decreasing pericardial compliance and the development of constriction, right ventricular filling pressures rise with subsequent flattening of the interventricular septum producing a characteristic ‘D’ shaped LV or even transient septal inversion to the left side (diastolic septal bounce) in early diastole^5,27^ (see Figure 7).

**FIGURE 8:**
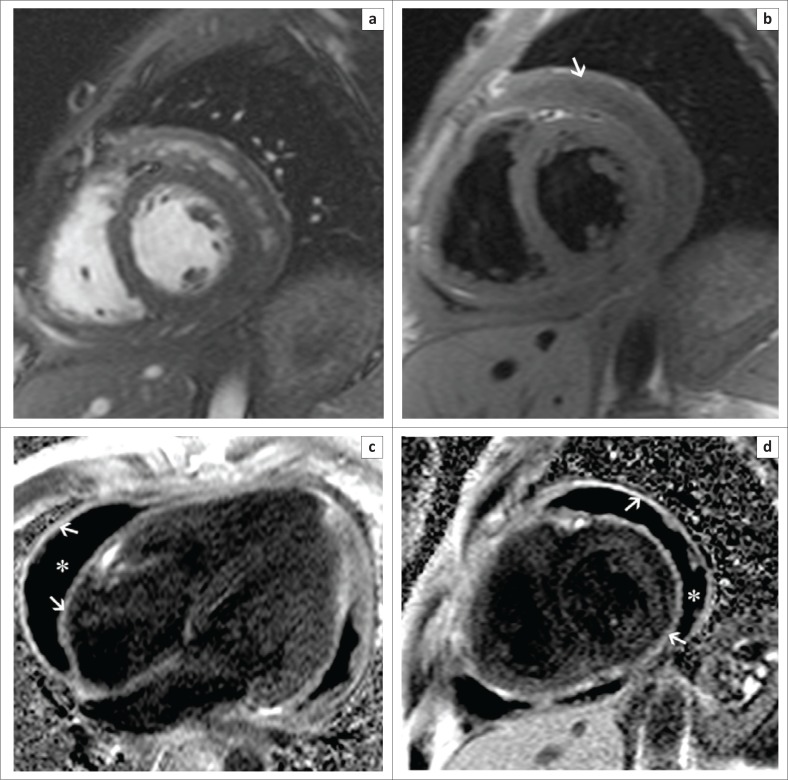
Short-axis SSFP (a) and T1-weighted (b) images demonstrate the presence of a large pericardial effusion (white arrow) and marked thickening of the visceral and parietal pericardium. Four-chamber (c) and short-axis (d) late gadolinium images show intense enhancement of the thickened visceral and parietal pericardium (white arrows) and the large hypointense pericardial effusion (white asterisk).

Pre- and post-contrast T1-weighted anatomic images allow accurate measurement of pericardial thickness and may show high signal areas of enhancement in keeping with active inflammation or fibrosis (see Figure 8). The detection of morphological abnormalities of the pericardium coupled with evidence of constrictive physiology is helpful in identifying patients who would benefit from surgical pericardiectomy and in guiding subsequent stripping procedures.

## Coronary artery disease

**FIGURE 9:**
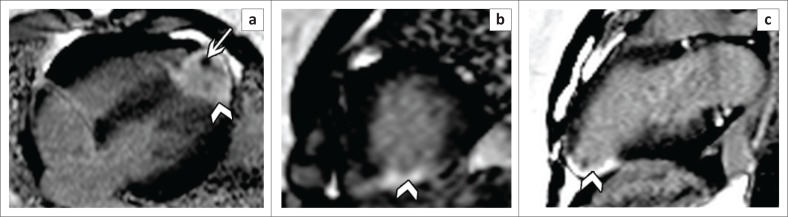
Four-chamber (a), short-axis (b) and 2-chamber (c) late gadolinium-enhanced images show a high signal transmural infarction of the LV apex (arrow heads) in an HIV-positive patient with spontaneous coronary artery dissection. The presence of a low signal intensity apical thrombus is also noted (white arrow).

The association between HIV and accelerated coronary artery disease (CAD) is well described and has been the focus of extensive study in recent years.^28^ Despite overall decline in all-cause mortality related to the introduction of ART, patients with HIV have an increased risk of acute myocardial infarction as compared with the general population.^29^ The reason for this is both complex and multifactorial, with HIV-mediated endothelin dysfunction and ART-related dyslipidaemia (particularly associated with protease inhibitor use) playing a role.^29^ Endothelial cells are thought to alter procoagulant, anticoagulant and fibrinolytic pathways *in vivo*, while underlying HIV-associated platelet dysfunction and background vasculitis likely contributes to the development of CAD.^30,31^ Furthermore, the chronic inflammatory state imposed by HIV infection is associated with an increased risk of spontaneous coronary artery dissection (see Figure 9). In addition to traditional Framingham risk factors, duration of HIV infection and lower CD4–CD8 ratios correlate with increased coronary arterial plaque burden.^32^

Despite the known relationship between HIV infection and accelerated atherosclerosis, the anticipated pandemic of CVD related to CAD has not materialised in sub-Saharan Africa. ‘The Heart of Soweto Study’ showed that, of the *de novo* manifestations of heart disease within the HIV-positive subset of the cohort (518 of 5328 cases), CAD was the primary diagnosis in only 14 patients (2.7%).^21^ In a cohort of 12 800 HIV-infected patients, Zhang et al. concluded that increasing age was associated with non-AIDS defining illnesses, including CVD.^33^ The widespread accessibility to ART may, in part, be responsible for the changing spectrum of diseases related to HIV, with an overall shift from AIDS-related illnesses to long-term age-related complications.^28^

Coronary angiography has been the gold standard for the assessment of the coronary arteries for several decades, offering high spatial and temporal resolution of coronary flow. A significant limitation has been its inability to provide information about the affected vessel walls themselves, preventing further study of plaque characteristics and vascular remodelling associated with ongoing or subclinical disease. Computed tomography is more recently recognised as a robust tool for the assessment of coronary anatomy and quantification of overall calcified and non-calcified plaque burden, but has limitations in its ability to offer further insights into the underlying properties of tissues being imaged. Ongoing advancements in high field strength coronary MR have shown great promise in accurately gauging the degree of coronary stenosis, allowing coronary vessel wall imaging, measurement of coronary blood flow velocities and further characterisation of non-calcified plaques using delayed enhancement imaging techniques.^32^ In a study of 35 HIV-positive patients, a significant increase in right coronary artery vessel wall thickness was found as compared with healthy controls.^34^ Another publication showed that the extent of local coronary epicardial adipose tissue was significantly related to coronary endothelial dysfunction as measured on coronary CMR.^35^ With the ongoing advances in the treatment for HIV and a steadily increasing life expectancy in infected individuals, accelerated CAD is likely to play a more significant role in HIV-related CVD over time, and novel CMR techniques offer insights that may broaden our understanding of this condition and inform future treatment strategies.

## Peripheral vascular disease

Human immunodeficiency virus-associated vasculopathy encompasses a wide spectrum of conditions including cerebrovascular disease, peripheral arterial thrombosis, arterial aneurysms and deep venous thrombosis. Its epidemiology remains incompletely characterised, with several described histological subtypes and a multifactorial aetiology.^36^ CMR techniques used to study this entity include measurement of reduced aortic distensibility, as demonstrated by cine acquisitions through various aortic planes and increased pulse wave velocity as calculated from phase contrast velocity encoded sequences. Both measures have been shown to be sensitive markers of reduced aortic elastic function, predictive of both future adverse cardiovascular events and mortality in several population-based studies. A study by Rider et al. found that treated HIV infection in patients without concurrent metabolic syndrome was associated with an 11% increase in pulse wave velocity and 14% regional reduction in aortic distensibility as compared with healthy controls.^37^ This effect was similar in HIV-negative patients with metabolic syndrome and additive in the subset of patients with both illnesses occurring concurrently.^37^

The effects of chronic inflammation and ART-associated lipodystrophy exert similar effects on the entire vascular system, with increased carotid artery intima-media thickness (C-IMT) on B-mode ultrasound shown to be predictive of future stroke and myocardial infarction. C-IMT, as measured by carotid CMR, has been shown to correlate well with sonographic measurements, with the added advantage of preserved resolution along the entire length of the examined arteries and ability to derive novel whole vessel parameters including plaque volume quantification, which may be useful as surrogate markers for longitudinal follow-up of cardiovascular risk in this subset of patients.^38^

## Human immunodeficiency virus-associated pulmonary arterial hypertension

The estimated prevalence of pulmonary arterial hypertension (PAH) in the HIV population is 0.5%, up to 2500-fold greater than that of the general population.^39^ The precise mechanism is uncertain, with both host and viral factors thought to contribute to the underlying pathogenesis. Furthermore, an increased propensity for thromboembolic disease or recurrent pulmonary infections with resulting pulmonary fibrosis likely contributes to the development of PAH and the high mortality associated with this condition.

The imaging findings of HIV-associated PAH are indistinguishable from those of primary PAH, with morphologic abnormalities observed including, dilatation of the pulmonary trunk, main pulmonary arteries and eventually the right ventricle (RV) and right atrium. Cine imaging is used for the quantification of RV indices (end systolic and diastolic volumes, EF and myocardial mass) and assessment of tricuspid regurgitation, following annular distortion related to RV dilatation.^40^ LGE has been noted at the superior and inferior LV or RV hinge-points in patients with PAH.^40^ The degree of enhancement has been shown to relate to RV volume and mass, raising suspicion that increased mechanical strain exacerbates structural deformation at the insertion points of the interventricular septum, resulting in architectural distortion and fibrosis of the myocardium in this region.^41^

## Myocardial steatosis

^1^H-MR spectroscopy (^1^H-MRS) is broadly accepted as a fast, accurate and reproducible means for the quantification of intra-cellular triglyceride content in the cytosol of non-adipose cells.^42^ Most commonly, a single volume of interest (voxel) is positioned over the interventricular septum, and myocardial spectra (with and without water suppression) are acquired at end-systole in several cardiac planes using either point-resolved spectroscopy (PRESS) or stimulated echo acquisition mode (STEAM) sequences. Subsequent analysis allows the expression of several myocardial lipid constituents (including triglycerides, saturated and unsaturated fatty acids) as a percentage of the overall tissue water content. Two recent studies using ^1^H-MRS have demonstrated increased myocardial lipid content in patients with HIV, adding weight to the hypothesis that morphologic alterations in myocardial tissue and possible lipotoxicity may underlie subclinical LV dysfunction.^11,43^

## Cardiac tumours and tumour mimics

Thrombi represent the most common cardiac mass, with stagnant blood flow in the setting of LV systolic dysfunction, myocardial infarction or aneurysms, and dilated cardiomyopathy coupled with a chronic inflammatory state predisposing to their formation. When large, these may be readily apparent on transthoracic echocardiography; however, suboptimal visualisation of the left atrial appendage and LV apex may compromise their detection with potentially disastrous clinical consequences. Additionally, despite good sonographic visualisation of the LV apex, layered mural thrombi may be difficult to differentiate from underlying myocardium.

In contrast, thrombi are readily detected on CMR, largely appearing as low intensity mass lesions that are incompatible with the normal myocardial contour or commonly noted intra-cardiac anatomical structures (e.g. crista terminalis, papillary muscles, moderator band and Eustachian valve) on standard cine SSFP sequences. The acquisition of early post-gadolinium inversion recovery sequences, typically 1–2 min following intravenous contrast administration, facilitates detection as the avascular thrombi appear hypointense in contrast to the enhancing blood pool and perfused myocardium.

**FIGURE 10:**
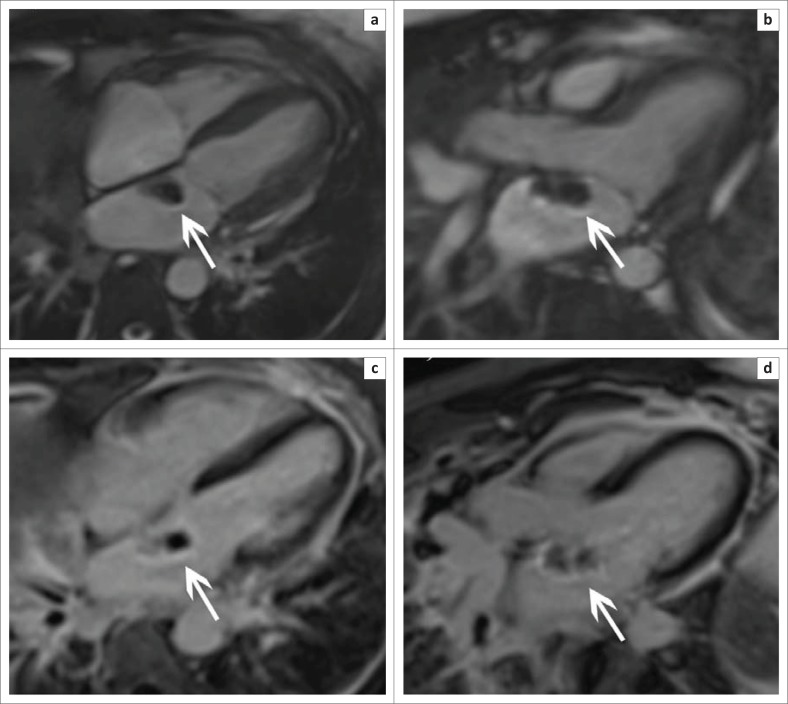
Four-chamber (a) and 3-chamber (b) SSFP sequences in a patient with known lymphoma show an isointense left atrial mass (arrows) arising from the inter-atrial septum. Corresponding late gadolinium-enhanced images (c and d) show heterogeneous enhancement of the mass.

There is a substantial increase in the risk of developing non-Hodgkin’s lymphoma in HIV-infected patients, with secondary involvement of the heart found frequently at post-mortem, thought to result from lymphatic, haematogenous or direct extension. Clinical presentation is often heterogeneous with patients remaining asymptomatic until the tumour produces significant mass effect or obstruction of cardiac chambers or great vessels,^44^ resulting in pulmonary or systemic embolisation^45^ or conduction abnormalities.^46^ Most cases are of the diffuse large B-cell subtype, presenting as a solid, ill-defined mass typically centred in the right atrium with infiltration along the epicardium and tricuspid annulus, with associated encasement, rather than invasion, of the coronary arteries.^47^ The relatively low likelihood of concurrent intratumoural haemorrhage or necrosis results in a homogenously hypo- or isointense mass on T1-weighted images, with variable LGE (see Figure 10).

Cardiovascular magnetic resonance thus enables comprehensive characterisation of cardiac masses, with cine imaging allowing characterisation of the size, shape and location of these lesions while allowing review of potential complications, including mass effect on adjacent coronary arteries and infiltration of surrounding structures, that may explain co-existing conduction defects and haemodynamic sequelae related to valvular dysfunction.

## Conclusion

Human immunodeficiency virus infection affects the entire cardiovascular axis through a complex interplay of multiple host, viral and treatment related factors. The availability of and access to ART have had a significant impact on improving the survival in patients with HIV or AIDS, and it is likely that over time, adverse cardiovascular events are likely to play an increased role in contributing to causes of morbidity and mortality in this subset of patients. CMR offers an opportunity to comprehensively assess the cardiovascular system, allowing detection of subclinical disease or detailed characterisation of known pathologies and informing subsequent clinical practice with the hopes of improving outcomes in affected individuals. Furthermore, the non-invasive and non-ionising nature of CMR make it a robust, flexible research tool well suited to longitudinal follow-up of patients and the development of novel imaging biomarkers that will serve to improve our understanding of disease processes, treatment effects and overall cardiovascular outcomes in HIV-infected individuals over time.
